# Retrospective comparison between a regular and a split-dose protocol of 5-fluorouracil, cisplatin, and mitoxantrone for the treatment of far advanced hepatocellular carcinoma

**DOI:** 10.1186/1471-2407-11-117

**Published:** 2011-03-31

**Authors:** Chau-Ting Yeh, Hui-Chin Chen, Chang-Mu Sung, Cheng-Lung Hsu, Chen-Chun Lin, Kuang-Tse Pan, Jeng-Hwei Tseng, Chien-Fu Hung

**Affiliations:** 1 Liver Research Unit, Department of Hepato-Gastroenterology, Chang Gung Memorial Hospital, Taipei, Taiwan; 2 Molecular Medicine Research Center, Chang Gung University, Taoyuan, Taiwan; 3 Graduate Institute of Nursing, Chang Gung University, Taoyuan, Taiwan; 4 Department of Hematology-Oncology, Chang Gung Memorial Hospital, Taipei, Taiwan; 5 Department of Diagnostic Radiology, Chang Gung Memorial Hospital, Taipei, Taiwan

## Abstract

**Background:**

In patients with advanced hepatocellular carcinoma (HCC), combination chemotherapy using 5- fluorouracil, cisplatin, and mitoxantrone (FMP) could achieve a response rate > 20%, but the beneficial effect was compromised by formidable adverse events. Chemotherapy given in a split-dose manner was associated with reduced toxicities. In this retrospective study, we compared the efficacies and side effects between a regular and a split-dose FMP protocol approved in our medical center.

**Methods:**

From 2005 to 2008, the clinical data of 84 patients with far advanced HCC, who had either main portal vein thrombosis and/or extrahepatic metastasis, were reviewed. Of them, 65 were treated by either regular (n = 27) or split-dose (n = 38) FMP and had completed at least one therapeutic course. The remaining 19 patients were untreated. Clinical parameters, therapeutic responses, survivals and adverse events were compared.

**Results:**

The median overall survival was 6.0, 5.2, and 1.5 months, respectively, in patients receiving regular FMP, split-dose FMP, and no treatment (regular versus split-dose group, P = 0.447; regular or split-dose versus untreated group; P < 0.0001). Patients receiving split-dose treatment had a significantly lower risk of grade 3/4 neutropenia (51.9 versus 10.5%, P = 0.0005). When the two treated groups were combined, the median overall survival was 10.6 and 3.8 months respectively for patients achieving disease control and progressive disease (P < 0.001). Cox proportion hazard model identified Child-Pugh stage B (hazard ratio [HR], 2.216; P = 0.006), presence of extrahepatic metastasis (HR, 0.574; P = 0.048), and achievement of disease control (HR, 0.228; P < 0.001) as independent factors associated with overall survival. Logistic regression analysis revealed that anti-hepatitis C virus antibody (odds ratio [OR], 9.219; P = 0.002) tumor size (OR, 0.816; P = 0.036), and previous anti-cancer therapy (OR, 0.195; P = 0.017) were significantly associated with successful disease control.

**Conclusions:**

Comparable overall survival was observed between patients receiving regular and split-dose FMP therapies. Patients receiving split-dose therapy had a significantly lower risk of grade 3/4 neutropenia. Positive anti-hepatitis C virus antibody, smaller tumor size, and absence of previous anti-cancer therapy were independent predictors for successful disease control.

## Background

Hepatocellular carcinoma (HCC) is the fifth most common solid malignancy and the third leading cause of cancer death in the world [1]. The major attributive factors include chronic hepatitis B virus (HBV) infection, chronic hepatitis C virus (HCV) infection and alcoholic liver diseases [2-4]. Early stage HCC can be cured by surgical resection, non-surgical ablation procedures or liver transplantation [5]. Although the 5-year survival rate reached over 50%, the recurrence rate remained high, reaching over 80% at the fifth year after surgical resection [5,6]. On the other hand, for patients with unresectable HCC, a standard therapy has yet to be established.

Transcatheter arterial chemoembolization (TACE) is believed to be beneficial in patients without main portal vein occlusion or extrahepatic metastasis. To date, no large-scale randomized study has been conducted to illustrate its beneficial effect. However, a profound difference in survival has been observed in small-scale case control studies as well as intent-to-treat studies [7-15]. Sorafenib has been shown to improve survival in unresectable HCC patients in two large-scale phase-III randomized controlled studies [16,17]. In the SHARP study, the median survival improved from 7.9 to 10.7 months and in Asia-Pacific study, the median survival improved from 4.2 to 6.5 months. Despite encouraging data, in both studies, it was shown that patients with extrahepatic metastasis could not significantly benefit from sorafenib therapy. In this subgroup of patients, systemic chemotherapy remained an option. Several phase-II trials using various chemotherapy regimens have been conducted for treatment of advanced HCC [18]. Among these regimens, only a few achieved response rates higher than 20%. When a single agent was used, doxorubicin could achieve a response rate of 32% in one study. When combination chemotherapy regimens were used, only three combinations have been shown to achieve a response rate greater than 20%. They are epirubicin + etoposide (39%), cisplatin + doxorubicin + 5-fluorouracil + alpha-interferon (PIAF, 26%), and mitoxantrone +5-fluorouracil + cisplatin (FMP, 27%) [19]. The response rate of FMP in the latter trial is consistent with that of another independent trial (23.8%) [20]. Despite substantial response rates, formidable side effect usually developed in a great proportion of patients, deterring the clinical application of these regimens in far advanced HCC. To date, no randomized controlled study ever conducted to clarify whether overall survivals can be prolonged with these agents. According to a large-scale study conducted in Taiwan, the overall survival for patients with far advanced HCC was 1.6 month [21], demonstrating the urgent for an effective therapeutic modality.

A recent advance in the strategy of chemotherapy is that chemotherapy can be performed in a split-dose manner with lower side effects [22]. This method, called metronomic chemotherapy, has now been widely adapted to various chemotherapy protocols. In our medical center, a regular and a split-dose protocol of FMP have been approved for the treatment of patients with advanced HCC. In this retrospective study, we compared the performance of these two protocols. The exploratory purpose of the present study was to investigate whether a split-dose FMP protocol was clinically acceptable for treatment of far advanced HCC and to justify the use of split-dose protocol in future clinical trials.

## Methods

### Patients

This was a retrospective study approved by the institutional review board of Chang Gung Memorial Hospital (IRB98-2109B). From June, 2005 to August, 2008, 4059 patients were diagnosed to have HCC in Chang Gung Medical Center. Of them, 894 (22%), 648 (16%), 1233 (30%), 462 (11%), and 822 (20%) patients received operation, radio-frequency ablation, TACE, chemotherapy, and no treatment, respectively. Of the 462 patients receiving chemotherapy, 286 patients were eligible and enrolled in various clinical trials, whereas in the remaining 176 patients, different approved single or combination anti-cancer agents were used. Of the 176 patients, 71 were diagnosed as far advanced HCC in the Department of Hepato-gastroenterology and had received FMP combination chemotherapy. Of these 71 patients, 65 had completed at least one course of FMP therapy and were included in this study. The remaining 6 patients were excluded due to failure to complete the first therapeutic course. Clinical parameters were recorded, including sex, age, HBV or HCV markers, alcoholism, ECOG performance status, clinical cirrhosis, portal vein thrombosis, tumor size, ascites, alpha-fetoprotein, previous treatment, distal metastasis, Child-Pugh classification, Okuda staging, Cancer of the Liver Italian Program (CLIP) score, treatment protocol used, and number of treatment courses. All patients included belonged to the advanced Barcelona Clinic Liver Cancer (BCLC) stage (stage C). Two different treatment protocols were available in our hospital, the regular and the split-dose protocols. The decision with regards to therapeutic methods was made after discussion between the attending hepatologists and the patients. HCC was diagnosed by biopsy or aspiration cytology. Alternatively, if tissue or cytology proof could not be obtained, HCC was diagnosed by high AFP levels (> 400 ng/mL) plus two dynamic image studies (dynamic computer tomography and angiography).

In this study, all 65 patients with far advanced HCC had main portal vein thrombosis and/or extrahepatic metastasis (not suitable for TACE); ECOG performance status 0-2; never received systemic chemotherapy except chemoembolization; adequate hematological data (hemoglobin > 9 g/dL; white blood cells > 2000 cells/mm^3^; neutrophils > 1000 cells/mm^3^; platelet count > 60,000 cells/mm^3^); adequate liver function (Child-Pugh classification A or B); and adequate renal function (serum creatinine within normal limits). The bilirubin levels were all ≦ 6.5 mg/dL except for two patients, who had bilirubin levels 23.4 and 24.5 mg/dL respectively because of tumor invasion to main biliary tree (Table 1).

**Table 1:** Patient characteristics

	Regular Group	Split-dose Group	Untreated Group	P (Regular vs. Untreated)	P (Split-dose vs. Untreated)	P (Regular vs. Split-dose)
Number of patients	27	38	19			

Age in years, average ± SD	61.0 ± 10.1	54.8 ± 14.9	62.8 ± 12.5	NS	0.049	NS

Gender (Male/Female)	21/6	31/7	16/3	NS	NS	NS

Etiology						

HBsAg (+)	19 (70.4%)	27 (71.1%)	10 (52.6%)	NS	NS	NS

Anti-HCV (+)	9 (33.3%)	11 (28.9%)	5 (26.3%)	NS	NS	NS

HBsAg (+)/anti-HCV (+)	1	1	0	-	-	

HBsAg (-)/anti-HCV (-)	0	1	4	-	-	

Alcoholism	11 (40.7%)	21 (55.3%)	7 (36.8%)	NS	NS	NS

ECOG performance status				NS^a^	NS^a^	NS^a^

0	9	19	7			

1	12	10	6			

2	6	9	6			

Diagnosis				-	-	-

Biopsy	5	10	4			

Cytology	9	9	0			

Imaging + alpha-fetoprotein	13	19	15			

Liver cirrhosis	27 (100%)	36 (94.7%)	17 (89.5%)	NS	NS	NS

Tumor size in cm, average ± SD	6.62 ± 3.81	7.99 ± 4.55	8.71 ± 3.42	NS	NS	NS

Alpha-fetoprotein in ng/mL, median (range)	448 (3-62208)	2338 (3-248421)	1491 (3 - 377218)	NS	NS	NS

Portal vein thrombosis	15 (55.6%)	22 (59.5%)	13 (68.4%)	NS	NS	NS

Metastasis	19 (70.4%)	19 (50%)	10 (52.6%)	NS	NS	NS

Lymph node	10	10	4			

Lung	8	10	7			

Bone	1	2	1			

Duodenum	1	0	0			

Adrenal gland	1	0	0			

Kidney	0	1	0			

Heart	0	1	0			

Inferior vena cava	0	1	0			

Child-Pugh stage						

A	16 (59.3%)	22 (57.9%)	10 (52.6%)	NS	NS	NS

B	11	16	9			

Bilirubin in mg/dL, average ± SD	1.44 ± 0.88	2.66 ± 5.20^b^	2.86 ± 2.36^c^	0.006	NS	NS

Previous therapy	18 (66.7%)	18 (47.4%)	No	-	-	NS

Percutaneous local ablation	3	2				

TACE	15	13				

Partial hepatectomy	3	3				

Radiotherapy	1	3				

Course of FMP chemotherapy received						

1	9 (33.3%)	25 (65.8%)	0	-	-	0.018

2	11	9	0			

3	2	2	0			

>3	3	1	0			

^a^Performance status was compared between ECOG 0 and ECOG 1+2 groups.

^b^Two patients had obstructive jaundice with bilirubin 23.4 and 24.5 mg/dL, respectively.

^c^One patient had obstructive jaundice with bilirubin 8.7 mg/dL.

NS, not significant (P > 0.05).

Another group of 19 patients with far advanced HCC, who had documented survival time, were also included. They all had main portal vein thrombosis and/or extrahepatic metastasis, ECOG performance status 0-2, and Child-Pugh functional class A or B. These patients did not receive any treatment following the diagnosis of HCC and were included as untreated controls. One of these patients had a bilirubin level of 8.7 mg/dL due to tumor invasion to biliary tree; otherwise all bilirubin levels were ≦ 6.5 mg/dL (Table 1).

### Treatment protocols

#### Regular protocol

5-FU was administered continuously via intravenous route at a dose of 450 mg/m2 on Days 1-5. Mitoxantrone was administered as an intravenous infusion at a dose of 6 mg/m2 on Day 1. Cisplatin was administered as an intravenous infusion at a does of 80 mg/m2 over 2 hours on Day 1 with standard hydration. The does used in the subsequent course was adjusted to the toxicities observed. Granulocyte colony-stimulating factor was given when neutropenia and/or leukocytopenia of Grade 3/4 were observed. The treatment was repeated every 4-6 weeks until reaching a maximum of 6 courses.

#### Split-dose protocol

The schedule was the same as the regular protocol one except that only a 1/2 dose of mitoxantrone and cisplatin were given on Day 1. On the ninth day, the biochemical and hematological data were checked. If the hematological, liver function, and renal function data were adequate, 1/4 dose of mitoxantrone and cisplatin were given on Day 9 and Day 10, respectively. The treatment was repeated every 4-6 weeks until reaching a maximum of 6 courses.

### Response and toxicity evaluation

The objective tumor response was assessed by computer tomography every 4-8 weeks after the beginning of FMP therapy and was evaluated according to the following criteria. Complete response (CR) was defined as the complete disappearance of all target lesions without any residual lesion. Partial response (PR) was defined as a > 50% decrease in tumor mass, without progression in any target lesion or appearance of a new lesion. Minor response (MR) was defined as a > 25% decrease in total tumor masses. Stable disease (SD) was defined as either a < 25% decrease or a < 25% increase of total tumor masses. Progressive disease (PD) was defined as a > 25% increase in total tumor masses. Clinical responders were patients achieving CR, PR or MR. Disease control was achieved in patients with CR, PR, MR or SD.

Adverse effects were evaluated according to the NCI Common Terminology Criteria for Adverse Events (CTCAE) version 3.0.

### Statistical analysis

Dichotomized data was expressed as ratios (%) and compared by use of Fisher's exact test; parametric data was expressed as mean ± standard deviation and compared by use of 2-sample t test; non-parametric data or data not in normal distribution was expressed as median (range) and compared by Mann-Whitney test. Overall survival was calculated from the date of treatment to the date of death or last follow-up. Time to disease progression was calculated from the date of treatment to the date of PD. In univariate analysis, the Kaplan-Meier method was used to estimate the survival probability and the log-rank test was used to compare the survivals between groups. Independent predictive factors affecting survival were analyzed by the Cox multivariate proportional hazards regression model. In this study, significant factors identified from univariate analysis were included for Cox proportional hazards analysis. Additionally, to follow the rule of 1 variable for 10 deaths, 6 most significant factors obtained from univariate analysis were selected for additional Cox model analysis. Independent predictors affecting disease control rate were analyzed by Logistic regression analysis. Only significant factors identified from univariate analysis were included. P < 0.05 was considered statistically significant. Statistical analysis was conducted by using SPSS (version 13.0).

## Results

### Basic clinical data in far advanced HCC patients included in this study

Of 84 patients included, 27 and 38 patients received regular and split-dose FMP therapy, respective, whereas 19 patients received no therapy. The basic clinical data are listed in Table 1. No statistical difference was found between the two treated groups in any of the pre-therapeutic clinical parameters. However, more patients in the split-dose group received only one course of FMP combination chemotherapy (regular versus split-dose groups, 9/27 (33.3%) versus 25/38 (65.8%); P = 0.018). When compared either of the two treated groups and the untreated group, it was found that patients in untreated group were borderline older than those in split-dose group (62.8 ± 12.5 versus 54.8 ± 14.9 years; P = 0.049). Additionally, patients in untreated group had higher bilirubin level than those in regular group (2.86 ± 2.36 versus 1.44 ± 0.88 mg/dL; P = 0.006). Such difference, however, was not observed between patients in the untreated and split-dose group (Table 1). Logistic regression analysis also failed to identify any clinicopathological factor significantly associated with the choice of either protocol. All except 3 responders and 2 patients with progressive diseases were followed till death.

### Clinical responses and associated factors

**Figure 1:**
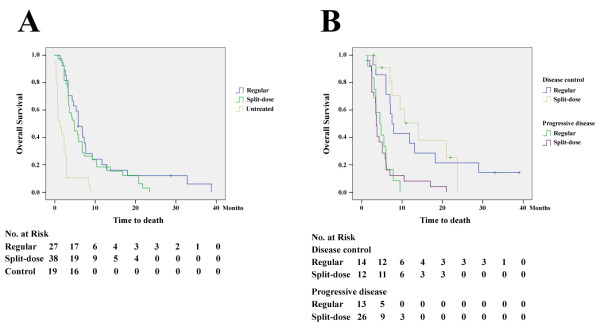
**Survival analysis of patients with advanced HCC treated by FMP**. (A) Overall survival in the two treated groups and the untreated group of patients. (B) Overall survival in the two groups of patients (regular and split-dose) achieving disease control and progressive diseases.

In patients on regular and split-dose protocols, 14/27 (51.9%) and 12/38 (31.6%) patients respectively achieved disease control (P = 0.127). The numbers of patients achieving CR, PR, MR, and SD were listed in Table 2. In the two groups of patients, the median overall survival was 6.0 and 5.2 months, respectively (P = 0.447) and the median time to disease progression was 3.1 and 2.2 months, respectively (P = 0.199). In contrast, the median overall survival was only 1.5 months in untreated patients (regular or split-dose versus untreated group; P < 0.0001; power = 99.6% [regular] and 99.3% [split-dose], respectively) (Figure 1A). In patients on regular protocol, the median overall survival was 7.5 and 4.5 months for patients achieving disease control and progressive disease, respectively (P = 0.002). In patients on split-dose protocol, the median overall survival was 14.0 and 3.6 months for patients achieving disease control and progressive disease, respectively (P < 0.001) (Figure 1B).

**Table 2:** Treatment outcome in patients receiving regular or split-dose FMP therapy

		Patients achieving disease control				
			
		Responders				Patients with progressive disease
				
Group	Total	CR	PR	MR	SD	
Regular	27	2	4	3	5	13 (48.1%)
Split-dose	38	0	4	5	3	26 (68.4%)

Regular vs. split-dose protocol: disease control rate, P = 0.127; response rate, P = 0.414.

Univariate analysis was conducted to identify factors associated with clinical responses, disease control, and overall survival (Table 3). Two factors were associated with higher clinical response rates: absence of previously anti-cancer therapy (44.8 versus 13.9%, P = 0.011) and completion of more than one course of FMP chemotherapy (45.2 versus 11.8%, P = 0.005). Seven factors were associated with higher disease control rates: age older than 60 years (57.1 versus 27.0%, P = 0.021), positive anti-HCV antibody (70.0 versus 26.7%, P = 0.002), negative serum HBV surface antigen (HBsAg) (63.2 versus 30.4%, P = 0.025), total tumor diameters less than or equal to 8 cm (54.8 versus 13.0%, P = 0.001), absence of previously anti-cancer therapy (55.2 versus 27.8%, P = 0.041), CLIP score 1 or 2 (55.6 versus 28.9%, P = 0.041) and completion of more than one course of FMP chemotherapy (64.5 versus 17.6%, P < 0.001).

**Table 3:** Univariate analysis for prognosis factors

Factors		No. of patients	Responder	P	Disease control	P	Median survival (months)	P
Sex	Male	52	13 (25.0%)	0.489	18 (34.6%)	0.114	5.6	0.374

	Female	13	5 (38.5%)		8 (61.5%)		7.4	

Age	≦ 60 years	37	9 (24.3%)	0.580	10 (27.0%)	0.021	5.1	0.050

	> 60 years	28	9 (32.1%)		16 (57.1%)		7.0	

anti-HCV	(-)	45	10 (22.2%)	0.229	12 (26.7%)	0.002	5.4	0.033

	(+)	20	8 (40.0%)		14 (70.0%)		7.6	

HBsAg	(-)	19	7 (36.8%)	0.364	12 (63.2%)	0.025	7.4	0.115

	(+)	46	11 (23.9%)		14 (30.4%)		5.6	

Alcoholism	(-)	33	11 (33.3%)	0.407	15 (45.5%)	0.450	6.0	0.611

	(+)	32	7 (21.9%)		11 (34.4%)		5.8	

ECOG	0	28	10 (35.7%)	0.267^a^	13 (46.4%)	0.446 ^a^	7.0	0.011^a^

	1	22	6 (27.3%)		11 (50.0%)		6.1	

	2	15	2 (13.3%)		2 (13.3%)		3.6	

Cirrhosis	(-)	2	0 (0%)	0.999	0 (0%)	0.513	11.5	0.830

	(+)	63	18 (27.7%)		26 (40%)		6.0	

Portal vein thrombosis	(-)	28	8 (28.6%)	0.999	12 (42.9%)	0.799	6.0	0.319

	(+)	37	10 (27.0%)		14 (37.8%)		5.5	

Tumor size	≦ 8 cm	42	15 (35.7%)	0.081	23 (54.8%)	0.001	7.1	0.001

	> 8 cm	23	3 (13.0%)		3 (13.0%)		4.2	

Ascites	(-)	41	10 (24.4%)	0.567	16 (39.0%)	0.999	6.0	0.014

	(+)	24	8 (33.3%)		10 (41.7%)		3.6	

Alpha-fetoprotein	≦ 10000 ng/mL	50	13 (26.0%)	0.743	21 (42.0%)	0.764	7.5	0.098

	> 10000 ng/mL	15	5 (33.3%)		5 (33.3%)		4.3	

Previous treatment	(-)	29	13 (44.8%)	0.011	16 (55.2%)	0.041	6.1	0.750

	(+)	36	5 (13.9%)		10 (27.8%)		5.2	

Extrahepatic metastasis	(-)	27	7 (25.9%)	0.999	8 (29.6%)	0.201	3.7	0.014

	(+)	38	11 (28.9%)		18 (47.4%)		7.1	

Child-Pugh	A	38	9 (23.7%)	0.414	13 (34.2%)	0.309	7.0	0.012

	B	27	9 (33.3%)		13 (48.1%)		3.8	

Okuda stage	I	27	10 (37.0%)	0.173^b^	14 (51.9%)	0.127^b^	7.0	0.004^c^

	II	28	5 (17.9%)		9 (32.1%)		5.1	

	III	10	3 (30.0%)		3 (30.0%)		3.1	

CLIP score	1 or 2	27	10 (37.0%)	0.172^b^	15 (55.6%)	0.041^b^	7.0	0.001^d^

	3	21	3 (14.3%)		5 (23.8%)		3,8	

	4 or 5	17	5 (29.4%)		6 (35.3%)		3.5	

Treatment groups	Regular	27	9 (33.3%)	0.414	14 (51.9%)	0.127	6.0	0.447

	Split-dose	38	9 (23.7%)		12 (31.6%)		5.2	

Courses	1	34	4 (11.8%)	0.005	6 (17.6%)	< 0.001	3.5	< 0.001

	> 1	31	14 (45.2%)		20 (64.5%)		7.9	

Responder	Yes	18	18 (100%)	NC	18 (100%)	NC	9.5	0.004

	No	47	0		8 (17.0%)		4.5	

Disease control	Yes	26	18 (69.2%)	NC	26 (100%)	NC	10.6	< 0.001

	No	39	0		0		3.8	

^a^patients with ECOG score 0 versus 1 and 2 combined.

^b^Patients with Okuda stage I versus those with II + III; patients with CLIP score 1 or 2 versus those with score > 2.

^c^Overall, P = 0.004; Okuda I versus II, P = 0.013; II versus III, P = 0.193.

NC, not calculated

^d^Overall, P = 0.001: CLIP 1 or 2 versus 3, P = 0.011; CLIP 3 versus >3, P = 0.432.

Factors associated with longer median overall survival were age older than 60 years (7.0 versus 5.1 months, P = 0.05), positive anti-HCV antibody (7.6 versus 5.4 months, P = 0.033), ECOG performance status = 0 (7.0 versus 5.1 months, P = 0.011), total tumor diameters less than or equal to 8 cm (7.1 versus 4.2 months, P = 0.001), absence of ascites (6.0 versus 3.6 months, P = 0.014), presence of extrahepatic metastasis (7.1 versus 3.7 months, P = 0.014), Child-Pugh classification A (7.0 versus 3.8 months, P = 0.012), Okuda stage I (7.0 versus 5.1 (stage II) and 3.1 (stage III) months, P = 0.004), CLIP score 1 or 2 (7.0 versus 3.8 (score 3) and 3.5 (score 4 or 5) months, P = 0.001), completion of more than one course of FMP chemotherapy (7.9 versus 3.5 months, P < 0.001), achievement of clinical responses (9.5 versus 4.5 months, P = 0.004) and achievement of disease control (10.6 versus 3.8 months, P < 0.001).

Cox proportion hazard model was used to evaluate the independent factors associated with overall survival. Of the 12 significant factors identified from univariate analysis, 11 were included for multivariate analysis. CLIP score was not included since all 4 components of this score had already been analyzed as individual variables. It was found that only Child-Pugh stage B (hazard ratio [HR], 2.216; 95% confident interval [CI], 1.257 - 3.905; P = 0.006), presence of extrahepatic metastasis (HR, 0.574; 95% CI, 0.330 - 0.995; P = 0.048), and achievement of disease control (HR, 0.228; 95% CI, 0.123 - 0.428; P < 0.001) were significantly associated with overall survival. The adjusted HR of other non-significant factors (P > 0.05) were age older than 60 years (1.194; 95% CI, 0.599 - 2.379), positive anti-HCV antibody (1.131; 95% CI, 0.489 - 2.614), ECOG performance status > 0 (2.005; 95% CI, 0.995 - 3.999), total tumor diameters > 8 cm (1.028; 95% CI, 0.430 - 2.460), absence of ascites (0.851; 95% CI, 0.344 - 2.104), Okuda stage I (1.387; 95% CI, 0.483 - 3.979), completion of more than one course (0.499; 95%, 0.245 - 1.013) and achievement of clinical responses (1.826, 95% CI, 0.600-5.555). In order to follow the rule of 1 variable for 10 deaths, Cox proportional hazard model was also performed by including only six most significant factors obtained from univariate analysis (P ≦ 0.011; Table 3). It was found that only achievement of disease control (HR, 0.158; 95% CI, 0.051 - 0.485; P = 0.001) remained significantly associated with overall survival.

Logistic regression analysis was used to evaluate independent factors predicting disease control. Six significant factors identified from univariate analysis were included for multivariate logistic regression analysis (CLIP score was not included). It was found that only anti-HCV antibody (odds ratio [OR], 9.219; 95% CI, 2.233 - 38.056; P = 0.002) tumor size (OR, 0.816; 95% CI, 0.674 - 0.987; P = 0.036), and previous anti-cancer therapy (OR, 0.195; 95% CI, 0.051 - 0.749; P = 0.017) were significantly associated with successful disease control.

### Adverse effect

The adverse effect found in patients receiving regular or split-dose protocol was compared (Table 4). Because different numbers of treatment courses were received among patients, all toxicity listed was assessed after the first course of chemotherapy. In patients receiving regular therapeutic protocol, the following grade 3 to 4 toxicities were observed in more than 10% of patients: leucopenia (37.0%), neutropenia (51.9%), thrombocytopenia (18.5%), renal toxicity (11.1%), bleeding (11.1%), and infection (11.1%). On the other hand, in patients receiving split-dose protocol, only grade 3 to 4 leucopenia (15.8%) and neutropenia (10.5%) were observed in more than 10% of patients. When the two groups were compared, patients receiving regular treatment protocol had a higher risk of grade 3/4 neutropenia (51.9 versus 10.5%, P = 0.0005; power = 96.2%). Furthermore, 3 patients treated with regular protocol had grade 4 (life-threatening) infection and 2 of them died. In contrast, 3 patients treated with split-dose protocol had grade 3 infection and all of them recovered after antibiotics treatment.

**Table 4:** Comparison of the maximum severity of toxicity between the two treatment groups

		NCI Common Toxicity Criteria Grade						
**Toxicity**	**Treatment group**	**0**	**1**	**2**	**3**	**4**	**Grade 3/4 (%)**	**P**

Leucopenia	Regular	2	7	8	8	2	10 (37.0%)	0.079
	Split-dose	19	10	3	6	0	6 (15.8%)	

Neutropenia	Regular	8	3	2	7	7	14 (51.9%)	0.0005
	Split-dose	26	3	5	4	0	4 (10.5%)	

Anemia	Regular	3	15	8	1	0	1 (3.7%)	> 0.1
	Split-dose	5	22	9	2	0	2 (5.3%)	

Thrombocytopenia	Regular	6	8	8	3	2	5 (18.5%)	> 0.1
	Split-dose	23	8	4	3	0	3 (7.9%)	

Nausea	Regular	14	11	2	0	0	0	> 0.1
	Split-dose	19	14	5	0	0	0	

Vomiting	Regular	23	2	2	0	0	0	> 0.1
	Split-dose	29	7	2	0	0	0	

Mucositis	Regular	22	0	5	0	0	0	> 0.1
	Split-dose	29	6	1	2	0	2 (5.3%)	

Diarrhea	Regular	18	8	1	0	0	0	> 0.1
	Split-dose	29	6	1	2	0	2 (5.3%)	

Skin rash	Regular	27	0	0	0	0	0	> 0.1
	Split-dose	37	1	0	0	0	0	

Fatigue	Regular	13	9	3	2	0	2 (7.4%)	> 0.1
	Split-dose	19	11	7	1	0	1 (2.6%)	

Renal	Regular	23	1	0	3	0	3 (11.1%)	0.067
	Split-dose	37	1	0	0	0	0	

Liver dysfunctiona	Regular	27	0	0	0	0	0	> 0.1
	Split-dose	35	0	3	0	0	0	

Bleeding	Regular	20	3	1	3	0	3 (11.1%)	> 0.1
	Split-dose	35	2	0	1	0	1 (2.6%)	

Infection	Regular	23	0	1	0	3	3 (11.1%)	> 0.1
	Split-dose	29	2	4	3	0	3 (7.9%)	

^a^Jaundice, Asterixis, and Hepatoencephalopathy were graded as grade 2, 3, and 4, respectively according to the criteria.

## Discussion

Before sorafenib was made available, TACE was the mainstay of treatment for eligible advanced HCC patients. Although no large-scale randomized study has been conducted, the palliative effect is well recognized. The median survivals in the literature ranged from 12.7 to 34 months, with most studies reporting a consistent median survival of 12.7 to 17 months [7-15]. It is now evident that sorafenib is effective in treating unresectable HCC patients after completion of the two phase-III studies [16,17]. However, in the SHARP study, most patients included were likely eligible for TACE. The median survival in the treatment group was 10.7 months. It is therefore questionable as to whether sorafenib should replace TACE in treating TACE-eligible patients. Additionally, in these studies, over 95% of patients included were Child-Pugh classification A, whereas in TACE studies, Child-Pugh classification B patients were usually included [8-15]. Finally, in both large-scale phase III studies, no statistical significance was observed between treatment and control groups in advanced HCC patients with extrahepatic metastasis [16,17]. Therefore, the use of systemic combination chemotherapy agents with higher response rates remains an option when treating HCC patients with distal metastasis. Of the most effective single or combination chemotherapy agents against HCC, anthracycline-based agents, such as doxorubicin, were notorious for causing cardiotoxicity. The cardiotoxicity might present as alteration of electrocardiac conductivity leading to arrhythmias, or as cardiomyopathy causing congestive heart failure. Along with other side effects, both doxorubicin and etoposide caused myelosuppression, which put patients at risk of severe infection. Furthermore, etoposide, albeit rarely, could cause acute myeloid leukemia. Among the combination agents that have been used for Phase II clinical trials, FMP appeared to have a consistent and acceptable response rate [19,20]. Although cardiotoxicity occurred less frequently, a high risk of grade 3/4 leucopenia and neutropenia remained. In this study, we discovered that by use of a split-dose protocol, the risk of neutropenia could be significantly reduced. As a result, no patient on the split-dose protocol died of neutropenia-related infection.

Although statistically insignificant, patients receiving split-dose protocol did have a slightly lower disease control rate as well as a shorter overall survival. However, when compared with the untreated group, the overall survival in split-dose group was still significantly prolonged (5.2 versus 1.5 months). Between the untreated and split-dose group, only borderline significantly older age was found in the untreated group (P = 0.049). This was due to the tendency that older patients mostly choose not to receive any treatment on the diagnosis of far advanced HCC. Interestingly, in univariate analysis (Table 3), it was found that older patients were more likely to achieve disease control (P = 0.021) and had a longer overall survival (P = 0.050). Therefore, the age difference between the untreated and split-dose groups did not affect our conclusion that the patients receiving split-dose FMP treatment had a better overall survival. Although the observation that older patients had a better prognosis seemed unreasonable, a large-scale study from Taiwan also indicated that HCC in younger patients had poorer prognosis [23]. We speculated that HCC in younger age was more invasive because cancer cells originated from younger patients had greater growth potential. Another seemingly possible bias is the short survival time in the untreated group (median survival 1.5 month). In Taiwan, most of the untreated terminal HCC patients were discharged against physician's advice and taken home. In this study, the untreated patients were included only when documented overall survival time was available. According to a large-scale study, the median overall survival time in terminal HCC patients was 1.6 month, similar to our observation [21].

Because of a short survival time in patients with far advanced HCC, overall survival is a better clinical endpoint for evaluation of treatment efficacy compared to progression free survival. In fact, of the 65 FMP-treated patients, 24 (40%) had a survival time less than 4 months. Additionally, the median overall survival time in the untreated patients was only 1.5 month.

A great majority of patients (65.8%) on the split-dose protocol received only one course of therapy (Table 1). In our hospital, the choice between the two therapeutic protocols and the number of therapeutic courses given were decided by the patients and attending doctors. As a result, patients choosing the split-dose protocol usually expressed more concern about the side effects of chemotherapy and therefore were reluctant to accept more than one course of therapy. It can be speculated that if most patients on the split-dose protocol had received more than one course of therapy, the survival could be further improved. The present data indicated that at least two courses of therapy should be given to achieve a better outcome. Clinically, physicians should encourage the patients to receive more than one cycle of therapy if no severe adverse reaction occurred. The present study is, however, limited by its retrospective nature. Although no significant association was found between the clinicopathological factors and the treatment choice between the two protocols, selection bias could still occur with a small sample size. Other unrecognized factors might affect the treatment choice but were not included in our analysis. Many patients with advanced HCC did not choose to receive any chemotherapy nor did they comply with a regular follow-up schedule in the outpatient clinic. Therefore, more comprehensive prospective, randomized study is needed before we can conclude that the split-dose protocol is indeed beneficial for terminal HCC patients.

It is surprising that Cox proportion hazard model identified extrahepatic metastasis as a factor beneficial for overall survival, albeit this finding has also been reported in a previous study [20]. In our hospital, however, only patients with main portal vein thrombosis and/or extrahepatic metastasis were eligible for systemic chemotherapy. Therefore, patients without extrahepatic metastasis in this study all had main portal vein thrombosis. This result could thus be interpreted as patients who had main portal vein thrombosis had a poorer overall survival compared with those who had distal tumor metastasis.

Another important finding in this study was that achieving disease control was more important than achieving partial response in terms of improving survival, since only achievement of disease control was included in Cox proportional hazard model as an independent factor. Finally, it was demonstrated that patients positive for anti-HCV were more likely to achieve disease control in both univariate and multivariate regression analysis. On the other hand, patients positive for HBsAg were less likely to achieve disease control in univariate analysis. The reason why HCV-related HCC responds better than HBV-related HCC is unclear. This might be attributed to different oncogenic pathways between HBV and HCV. For example, frequent integration of HBV-DNA into chromosome of non-cancerous liver tissue in HBV associated HCC were reported, resulting in multi-focal clonal population of hepatocytes [24]. As a result, HBV associated advanced HCC might have a more complex composition of multi-clonal cancer cells and thus responded less well to chemotherapy.

## Conclusion

In conclusion, we discovered that by using a split-dose protocol, the toxicity of FMP combination chemotherapy could be drastically reduced with no significant alteration of the overall survival. FMP chemotherapy was more likely to achieve disease control in patients with positive anti-HCV, smaller tumor size, and previously not having treated with anti-cancer therapy.

## Competing interests

The authors declare that they have no competing interests.

## Authors' contributions

C-T Y, C-L H, H-C C and C-C L designed the study and analyzed the clinical data. C-T Y and C-M S involved in drafting the manuscript and revising it critically for important intellectual content. K-T P, J-H T, and C-F H interpreted and analyzed the radiological imaging data. All authors have read and approved the final manuscript.

## Pre-publication history

The pre-publication history for this paper can be accessed here:

http://www.biomedcentral.com/1471-2407/11/117/prepub
